# Observations on Delivery in Difficult Cases of the Presentation of the Shoulder of the Fœtus; and Where One or Both Arms Present along with the Head

**Published:** 1802-09-01

**Authors:** James Barlow

**Affiliations:** Blackburn, Lancashire


### *Observations on Delivery in difficult Cases of the Presentation of the Shoulder of the Fœtus; and where one or both Arms present along with the Head; communicated by* Mr. JAMES BARLOW, *of Blackburn, Lancashire.*

WITHOUT attempting to explain the caufe of preternatural prefentations of the Fœtus, I will content myfelf with briefly offering a few remarks on this branch of obftetric knowledge, through the medium of your much efteemed and valuable Journal.

On taking a curfory view of the hiftory of Midwifery, it is evident that it was the practice of the antients in every fpecies of prefentation, excepting the head, to reverfe the part prefenting, and fubftitute the head in its place, and various uncouth expedients were had recourfe to, to effectuate this fubverfion of the foetus.

We find Hippocrates, who lived 460 years before the Chriftian era, and from which period the moft authentic improvements in the obftetric art may be dated, recommending for this purpofe, among other abfurd cuftoms, to fhake and roll the woman on the bed; and if this method fail, he further advifes to difmember the foetus, and extract it with inftruments; for he allows no delivery to be natural, except that of the head firft prefenting: And this opinion was fo generally received, that Pliny eftablifhed it in his time as a maxim, that the order of Nature is to enter into the world by the head; and the cuftom to go out of it by the feet. Ariftotle and Galen were of the fame opinion, and regarded the delivery by the feet as foreign to Nature. Amongft others who embraced the fame notion are Mercurialis, Galeatius, James Rüeft, Eucharius * Rhodion, Mercatus, Varundus, and Perdulcis. Though the opinion of thefe authors was generally adopted, yet the method of turning and delivering by the feet was difputed for the fpace of fixteen centuries; and this doctrine, with fome other vague improvements in the art, was the practice chiefly perpetuated till the time of Ambrofe Parey, who, about the year 1575, publifhed his work on the Generation of Man, wherein he deviates from moft of his predeceffors, and rejects the prepofterous cuftoms eftablifhed in thofe times, and expressly points out the great improvement in the obftetric art, of turning the foetus, and delivering by the feet in all preternatural prefentations †.

About this period this important art began to emerge from obfcurity; and, amongft the advocates for the fame mode of de-

* The practice adopted by this author, who publifhed a work intitled, The Garden of Rofes, for pregnant ladies and midwifes, in the year 1551, was generally followed during that century, without any effential improvement.

† Parey may juftly be ranked amongft the firft who alfo introduced the practice of turning in cafes of uterine hæmorrhage; though this work is filent on that head, yet his pupil, Guillemeau, acknowledges this when attending Mad. Simon, daughter to Ambrofe Parey. Louifa Burgeois, who publifhed a Treatife on Midwifery about the year 1609, mentions this mode of delivery in cafes of flooding during pregnancy. See Chapter 4th and 5th.

The celebrated Puzos has alfo illuftrated this fubject with much ingenuity. See the Memoirs of the Royal Academy of Surgery at Paris.

livery, promulgated by Parey, for example, are Celfus, Ætius, Mofchion, Paul d'Egina, Serapion, Avicenna, Philomenes, Marinello, and Albucafis. Guillemeau, who was pupil to Ambrofe Parey, after much experience, adopted the method of turning and delivering by the feet; but the practice met with much oppofition at different periods, and never became general till the time of Mauriceau; fince which epoch, the art of midwifery has been gradually advancing towards perfection; and the lateft authors have furnifhed us with precepts ftill better calculated to furmount impending difficulties. What chiefly renders the prefentation of the fhoulder the more perplexing, is, when the liquor amnii has been long evacuated, and that extremity wedged in the brim of the pelvis by the repeated and violent action of the uterus upon the body of the child. In this fituation, the accoucheur fhould make himfelf early acquainted with the ftate of the os uteri, and if the orifice of that vifcus be fo far dilated as to admit the operator's hand, and the pains not ftrong, the fituation of the fœtus fhould then be afcertained with as much accuracy as poffible; and if the uterus prefents no inordinate obftacle, delivery may then be accomplifhed, by gently infinuating the hand along the breaft of the child and bringing down the feet, thus finifhing the delivery according to the rules laid down by authors. If, however, on the difcharge of the waters, the pains fhould continue without remiffion, delivery will be more hazardous, and the difficulty attendant on turning the fœtus will be in proportion to the violent and irregular contractions of the uterus; and during the prefence of the pains, the accoucheur fhould be cautious of exerting much force in attempts to introduce the hand or raife the fhoulder, left a rupture of that organ be the confequence. In this ftate, a quantity of blood taken from the arm of the mother, and a clyfter injected up the rectum, containing about 100 or 120 drops of tinct. opii, will generally moderate the violence of the uterine action fufficient to allow the introduction of the hand for the purpofe of extracting the child; yet, notwithftanding our utmoft efforts, when delivery has been protracted beyond due time, and the waters long evacuated, the child generally falls a victim eventually, even though the pelvis be well formed, and every means ufed which the art can fuggeft. In fome cafes, though the fhoulder of the fœtus originally prefented alone, yet when the orificium uteri is become confiderably dilated, the arm will, by the efforts of the uterus, be forced down into the vagina, and the hand be protruded at the os externum. In this ftate it has been recommended by fome authors, to amputate or twift off the arm of the fœtus at the fhoulder, in order to facilitate the introduction of the hand of the accoucheur; but but this trifling impediment feldom or ever calls for fuch inhuman interference, and the records of obftetric practice furnifhes us with but too many inftances of fuch wanton barbarity or ignorance.

Chapman relates a cafe of this kind, where the fœtus was fuppofed by the accoucheur to be dead, and who, after having one arm amputated whilft in the vagina uteri, afterwards lived to be a man. More examples might be adduced from authors, of children being unneceffarily difmembered, and a few of this defcription have fallen under my own cognizance, but it is unneceffary to recount fuch leffons of cruelty, as they can ferve no good purpofe to direct our future practice.

It fometimes happens, when the fhoulder of the fœtus has become wedged in the fuperior aperture of the pelvis by the continued force of the uterus, in fuch a manner that the hand of the operator cannot pafs by the prefenting fhoulder; to encounter this obftacle, it has been recommended to force or raife the child by inftruments. Dr. Burton made ufe of what he calls a crutch for this purpofe; and the impellens of Albucafis is intended to produce the fame effect, by raifing the prefenting part of the child; but when the pains have abated, this may be furmounted with greater facility and fafety by means of the operator's hand properly applied in the arm-pit of the child, and raifing the fhoulder towards the fundus uteri, and thus perfevering gradually in this manner, the hand will gain admiffion to the feet, and delivery be effected without the ufe of inftruments. It rarely happens, after every means has been ufed, that the fhoulder becomes impelled by the force of the uterus fo far in the pelvis, that it is utterly impoffible to introduce the hand or raife the fhoulder above the brim of the pelvis; and when this has been the cafe, the powers of the uterus have in fome inftances accomplifhed the delivery by a fpontaneous evolution of the fœtus: A cafe of this defcription occurred to me about ten years ago, the particulars of which I will briefly relate. The woman was at the full period of her firft uterine geftation; I was called to her on the Friday evening, after a midwife had been with her all that day, and part of the preceding night; on examination, I found the arm prefenting, and the hand of the fœtus protruded confiderably out at the os externum, and very much fwoln; the waters were evacuated early on the day before, the pains were ftrong, and the orificium uteri was confiderably dilated. I made an attempt to introduce my hand, but the uterine efforts were fo forcible, that I could obtain no advantage: About twelve o'clock the fame evening, I gave her forty drops of * tinct opii, but without perceiving any

* Would not the Digitalis, either given alone, or combined with opium, be of fervice in abating the immoderate action of the uterus?

abatement of the pains; the ſame quantity was adminiſtered in about two hours from taking the firſt doſe, but without producing the deſired effect. I again made another attempt to introduce my hand, but with no better ſucceſs than before, for the pains returned with redoubled force and frequency, and the action of the uterus was now ſuch, as to forbid any further attempts to turn the fœtus; the ſhoulder of the child now became forcibly preſſed upon the perineum; ſhe continued in this extreme miſery till the following evening, when the force of the uterus was ſo great, that I expected a rupture of that organ would every moment take place. Being once more induced to aſcertain the nature of the caſe, I was agreeably ſurpriſed to find the preſenting arm of the fœtus retracting, and the breech ſoon ſupplied the place of the ſhoulder, and was expelled in a few minutes by the powers of the uterus, after which the ſhoulder and head were extracted, and the placenta immediately followed the exit of the child, which was dead. Notwithſtanding the woman's ſevere ſufferings, ſhe had a ſpeedy recovery.

Though the ſpontaneous evolution of the fœtus will, under certain circumſtances which are concealed* from us, occaſionally take place, and granting to the inherent efforts of the uterus its full force, there is reaſon to believe, that were every caſe of the preſentation of the ſhoulder left entirely to the power of Nature, many lives would be thereby ſacrificed, which by a timely and well regulated interference, might be preſerved: it is I preſume only a very rare occurrence when the evolution terminates the delivery, and that almoſt always with deſtruction to the fœtus; and is it not a little ſurpriſing, that the diſcovery of ſuch a phenomenon ſhould have eſcaped the notice of all authors before the time of Dr. Denman, more particularly during that period when the art of turning and delivering by the feet was unpractiſed? There are few caſes of the preſentation of the ſhoulder, and when the pelvis is well formed, where delivery may not be accompliſhed with perfect ſafety by turning either immediately after the evacuation of the waters, or by delaying the attempt till the action of the uterus has abated, after having moderated its efforts by adminiſtering the abovementioned

* In the 38th chapter of the book of Geneſis, there is a curious account of (Tamar) bringing forth living twins; the firſt which preſented with the arm ſo far protruded that a ſcarlet thread was tied upon the hand, was the latter of the two fœtuſes which came forth. How this reverſion could be I will not offer to explain; but it appears that the midwife was a little ſurpriſed at the occurrence, for ſhe exclaimed, "How haſt thou broken forth! this breach be upon thee!" Which expreſſion gives reaſon to conclude, that ſuch an event was uncommon in her practice; and alſo that ſhe was not in the habit of turning and delivering by the feet.

mentioned medicines. It has been the practice with fome accoucheurs, in cafes of great difficulty, to fever the head from the body of the foetus with the blunt hook, or an inftrument refembling the crotchet, with a fharp ridge on the concave part of the bend, and fimilar to the one defcribed by Celfus. This author, however, differs in one refpect from the mode of delivery adopted by others in the ufe of this inftrument; for he directs to bring away the head firft, and after that the reft of the body. How far either of thefe methods of operating with hooks, and beheading the fœtus whilft in the uterus, may be warrantable, I cannot abfolutely pretend to determine, having never put them in practice, but am inclined to believe, that where an inftrument fuch as the crotchet can be ufed with fafety to the mother as high in the pelvis or uterus as to effect a feparation of the head from the body of the fœtus, the hand of the accoucheur might with equal facility and fafety be conducted into that organ, and the fœtus turned by bringing down the feet; or, if this fimple expedient fail, it may then be requifite to fix a noofe on one or both feet, and pull thereat in a reciprocal or wriggling direction with one hand, whilft the other is employed in raifing the fhoulder or body of the child towards the fundus uteri. By acting in this manner, and with due perfeverance, the fituation of the fœtus will be reverfed; and if the pelvis be well formed, delivery may be thus accomplifhed without having recourfe to any inftrument for the purpofe of difmembering the fœtus. If, on the other hand, the apertures of the pelvis are fo much contracted that the head of a mature fœtus cannot enter; or, in other words, when it appears manifeft to the accoucheur that the fuperior ftrait of the pelvis does not exceed three inches in diameter, then every attempt to turn the fœtus as above directed would be ufelefs; and no other mode of effecting delivery, under thefe circumftances, appears more eligible than by the ufe of the inftruments generally employed in embryulcia †.

I come now to treat on that fpecies of preternatural prefentation where one or both arms prefent, and the head either refting on fome part above the brim, or advanced along with either of the fuperior extremities into the pelvis. The mode of delivery fanctioned by authors in this divifion of prefentation, appears involved in fome degree of ambiguity; for little variation of practice has been adopted, whether the prefentation

† Vide Select Cafes in Midwifery, by Dr. James Hamilton; alfo Medical and Phyfical Journal, No. XL. and Heifter's Surgery.

tion of the hand or hands at the brim of the pelvis, or that of one or both arms defcended low in the vagina, the fame plan of delivery in every ftate, however diffimilar the cafe may be, feems to be invariably purfued. I conceive there requires much difcrimination as well as variety of practice neceffary to be adopted, according to different fituation and ftages in which the fœtus is found to prefent under this diftinction of preternatural prefentations.

If the accoucheur has the management of a cafe of this kind from the time of the difcharge of the liquor amnii, and the hand of the fœtus is afcertained to prefent, and the head can be brought into the axis of the pelvis, it fhould be effected as early as the ftate of the os uteri will admit, and the reduction of the hand in this ftate of the cafe may be frequently accomplifhed by pufhing it up and fupporting it at the brim of the pelvis till the head becomes engaged in the fuperior aperture: if the other hand fhould protrude, it may be encountered by a fimilar expedient. If thefe attempts prove ineffectual, I would recommend the accoucheur to introduce a piece of fponge or other foft fubftance along the cavity of the pelvis during the abfence of a pain, and wedge or reftrain the prefenting hand or hands above the fuperior ftrait, till the head has cleared the brim of the pelvis. It may be alleged, that the introduction of fuch fubftance into the cavity of the pelvis, may obftruct the exit of the child; but this, on reflection, cannot be admitted as an objection, for the fubftance introduced will either be pufhed forwards along with the head, or otherwife be lodged above the brim of the pelvis, and in either cafe will prove no obftacle to delivery. It will be evident that I here allude only to thofe cafes where one or both hands prefent, and not where the fore arm is advanced to the os externum, and the head engaged in the cavity of the pelvis. I have in a few cafes fucceeded by replacing the hand as above directed; and in one inftance of the defcent of the funis umbilicalis along with the head, preferved the life of the fœtus by pufhing up the defcended funis out of the way of compreffion, and introduced a quantity of cotton wool in fuch a manner as to plug up the paffage on that fide of the head where the funis protruded. The fame mode of proceeding may be adopted with advantage when the breech prefents together with the funis, if the pulfation be perceptible in the cord.

When the head of the fœtus has defcended fo far in the pelvis as to become wedged in that tube, and either the funis or one or both arms of the fœtus protrude therewith, then every attempt to turn the child fhould be abandoned, and the event of the cafe be left to the efforts of Nature; and if thefe fail to accomplifh the exit of the child, then the ufe of the lever becomes comes admiffible, and delivery will, even under thefe circumftances, be fometimes crowned with fuccefs; for, by purfuing an adverfe plan of delivery, the child in all probability would be facrificed, and the mother's life endangered; it is then much fafer to leave the event to Nature; and if the efforts of the uterus be infufficient to effect delivery, even when affifted by the lever, recourfe may then be had to the crotchet. When one arm only prefents, and the head advances in a proper direction, there appears lefs risk both to the mother and fœtus by not either attempting to reduce the hand or reverfing the fœtus; for one arm prefenting with the head in a well formed pelvis, will feldom oppofe any material obftacle to delivery more than what may be eafily obviated by the ufe of the lever; but when both fuperior extremities prefent, and the head is advanced in the pelvis, the cafe wears a more unfavourable afpect; and this in proportion to the capacity of the pelvis, and rigidity of the foft parts; and yet, when the reduction of the parts prefenting with the head cannot be effected without exerting much force, and the head is become engaged, and the pains ftrong, Nature will frequently be able to complete her own work with fafety to both mother and child without manual affiftance. As a proof of what I am advancing, I have attended two cafes of this defcription, when both arms prefented with the head of the fœtus, and the delivery was terminated without harm either to mother or child, in both inftances, by the efforts of Nature alone. Another cafe of the fame kind I was witnefs to, where the accoucheur, with great difficulty, finifhed the delivery by turning: the child was dead, and the mother had a very narrow efcape in confequence. The difficulty and danger of reducing the hand or arm of the fœtus will be in proportion to the action of the uterus; and the great obftacle which generally oppofes the introduction of the hand, is the contraction of the cervix uteri, and not the protruded part of the fœtus, as is too generally fuppofed; notwithftanding, the reduction of the fuperior extremities, when advanced with the head into the cavity of the pelvis, is frequently impracticable, and may fometimes prove dangerous both to the mother and fœtus when much force is ufed with this view; or, what is ftill more hazardous, by turning and delivering by the feet; nor does the practice of turning in this fpecies of prefentation become effentially neceffary; neither does the reduction of the hand (when the pelvis is well formed) except it can be accomplifhed before the head be engaged in the fuperior aperture. If the accoucheur be called early, and before the membranes are ruptured, he will fometimes be able to diftinguifh the fingers of the fœtus before their rupture takes place. In this fituation, if the uterus be fufficiently ciently dilated, it may be prudent to introduce the hand and rupture the membranes, rather than leave this evacuation to a ſpontaneous event: this being done, the arm of the fœtus will generally, if not prevented, be forced down by the efforts of the uterus; the accoucheur ſhould then immediately endeavour to obviate, if poſſible, by engaging the head and retaining the hand of the fœtus above the brim of the pelvis: if this cannot be accompliſhed, and either the funis or arm of the fœtus protrude, the operator's hand ſhould then be conducted to the feet of the child, and delivery terminated by turning.

Notwithſtanding all theſe efforts, the limits of our art are too often ſuch as do not altogether furniſh us with reſources in this ſpecies of birth, ſufficient to ſurmount every obſtacle adequate for the preſervation of the fœtus; neverthelеſs, if theſe ſcattered fragments ſhould be the means of ſaving a ſingle life, I ſhall not conſider the little time beſtowed on the ſubject ill employed.

*Auguſt* 2, 1802.

